# A clinical study to optimise a sand fly biting protocol for use in a controlled human infection model of cutaneous leishmaniasis (the FLYBITE study)

**DOI:** 10.12688/wellcomeopenres.16870.1

**Published:** 2021-06-30

**Authors:** Vivak Parkash, Helen Ashwin, Jovana Sadlova, Barbora Vojtkova, Georgina Jones, Nina Martin, Elizabeth Greensted, Victoria Allgar, Shaden Kamhawi, Jesus G. Valenzuela, Alison M. Layton, Charles L. Jaffe, Petr Volf, Paul M. Kaye, Charles J. N. Lacey

**Affiliations:** 1 York Biomedical Research Institute, Hull York Medical School, University of York, York, N.Yorks, YO10 5DD, UK; 2 Department of Infection and Tropical Medicine, Sheffield Teaching Hospitals NHS Foundation Trust, Sheffield, UK; 3 Department of Parasitology, Charles University, Prague, Czech Republic; 4 School of Social Sciences, Leeds Beckett University, Leeds, UK; 5 Peninsula Medical School, University of Plymouth, Plymouth, UK; 6 Laboratory of Malaria and Vector Research, National Institutes of Health, Rockville, MD, USA; 7 Department of Microbiology and Molecular Genetics, The Hebrew University – Hadassah Medical School, Jerusalem, Israel

**Keywords:** Controlled human infection models; leishmaniasis, vaccines, sand flies; public engagement, focus groups

## Abstract

**Background:** Leishmaniasis is a globally important yet neglected parasitic disease transmitted by phlebotomine sand flies. With new candidate vaccines in or near the clinic, a controlled human challenge model (CHIM) using natural sand fly challenge would provide a method for early evaluation of prophylactic efficacy.

**Methods** *: *We evaluated the biting frequency and adverse effects resulting from exposure of human volunteers to bites of either *Phlebotomus papatasi* or *P. duboscqi*, two natural vectors of *Leishmania major*. 12 healthy participants were recruited (mean age 40.2 ± 11.8 years) with no history of significant travel to regions where *L. major*-transmitting sand flies are prevalent. Participants were assigned to either vector by 1:1 allocation and exposed to five female sand flies for 30 minutes in a custom biting chamber. Bite frequency was recorded to confirm a bloodmeal was taken. Participant responses and safety outcomes were monitored using a visual analogue scale (VAS), clinical examination, and blood biochemistry. Focus groups were subsequently conducted to explore participant acceptability.

**Results:** All participants had at least one successful sand fly bite with none reporting any serious adverse events, with median VAS scores of 0-1/10 out to day 21 post-sand fly bite. Corresponding assessment of sand flies confirmed that for each participant at least 1/5 sand flies had successfully taken a bloodmeal (overall mean 3.67±1.03 bites per participant). There was no significant difference between *P. papatasi* and *P. duboscqi *in the number of bites resulting from 5 sand flies applied to human participants (3.3±0.81 vs 3.00±1.27 bites per participant; p=0.56) *. * In the two focus groups (n=5 per group), themes relating to positive participant-reported experiences of being bitten and the overall study, were identified.

**Conclusions: **These results validate a protocol for achieving successful sand fly bites in humans that is safe, well-tolerated and acceptable for participants.

**Clinicaltrials.gov registration: **NCT03999970 (27/06/2019)

## Introduction

The World Health Organisation (WHO) has identified several neglected tropical diseases postulated to be vaccine preventable but where progress in vaccine development has been limited ^1^. Included amongst these are the leishmaniases, a group of diseases caused by different species of *Leishmania* parasites that affect over 100 million people across 98 countries ^2, 3^, with an estimated 1 billion living in endemic areas at risk of infection worldwide ^4, 5^. Although the majority of cases of leishmaniasis affect the skin (the tegumentary leishmaniases, including localised cutaneous leishmaniasis, mucosal leishmaniasis, diffuse cutaneous leishmaniasis, disseminated cutaneous leishmaniasis and post kala azar dermal leishmaniasis), some species of *Leishmania* cause visceral leishmaniasis (VL or kala azar) affecting the internal organs and leading to death if untreated. Up to 18,700 deaths from VL occurred in 2019, a reduction from past decades that is in part attributed to the ongoing elimination campaign in South Asia ^4, 6, 7^. Phlebotomine sand flies (Diptera: Phlebotominae), mostly belonging to the genera *Phlebotomus* and *Lutzomyia*, are biological vectors of *Leishmania,* and some exhibit close evolutionary relationships with specific *Leishmania* species ^8, 9^.

Significant challenges in leishmaniasis control remain including a lack of effective treatments and drug resistance ^10^, a poor understanding of infection reservoir dynamics ^11, 12^ and the limited impact of vector control programmes ^13^. The availability of an effective vaccine would have a major impact on health and economic development in low-and-middle income countries (LMICs) where leishmaniasis is endemic, particularly on transmission and population incidence, as well as potentially leading to elimination ^14^. To-date no human vaccine for leishmaniasis has been licensed, although several vaccines are being developed ^15– 17^. Numerous barriers limit the development of such vaccines, including resource allocation to leishmaniasis research, limited translational application of animal models, and lack of effective correlates of protection.

For several diseases where there is an urgent need for a vaccine, controlled human infection models (CHIM) have been proposed as a mechanism for efficiently and cost-effectively evaluating new vaccines and therapies ^18, 19^. In such models, healthy participants are deliberately infected with the pathogen of interest and at the end of the observation period, interventions are used to curtail the infection. Over the last half century, structured evidenced-based and ethical approaches to CHIM studies have gained traction. CHIM models have been developed for a range of viral, bacterial, protozoan and helminth diseases ^20^, including malaria ^18^, influenza ^21^, norovirus ^22^, dengue ^23^, *Streptococcus pneumoniae* ^24^ and schistosomiasis ^25^. More recently CHIM studies have been proposed as a means of testing potential severe acute respiratory syndrome coronavirus 2 (SARS-CoV-2) vaccines in order to control the coronavirus disease 2019 (COVID-19) pandemic ^26, 27^. In some CHIM studies, including with malaria vaccines ^18^, the natural vector has been used to facilitate infection, and this may be particularly important when the process of vector transmission facilitates infection or alters immune responses in a manner not mimicked by needle challenge ^28– 30^.

Multiple arguments support the notion that the leishmaniases are vaccine preventable diseases including demonstrable immunity following infection and self-cure ^16, 31^. “Leishmanization” in areas endemic for cutaneous leishmaniasis involved the inoculation of live virulent parasites into cosmetically less conspicuous areas, typically on the buttock, to prevent subsequent lesion development at more stigmatizing sites. Whilst a testament to the ability of prior infection to generate protective immunity, this practice was discouraged due to ethical concerns ^32, 33^. Leishmanization nevertheless provided the basis for a proof of concept *Leishmania* CHIM in Iran, conducted in 2005, that demonstrated using needle challenge, that a reasonable take rate, self-healing of lesions and subsequent protection could be achieved using parasites expanded under GMP conditions ^32, 33^. This approach, however, was not pursued further. The development of new candidate vaccines for leishmaniasis, in ^34– 36^ or near to clinical trial ^37– 39^, provides the impetus to re-evaluate and update the previous CHIM model. Recent findings that broaden the understanding of the challenges of a *Leishmania* CHIM are also an important consideration, including the importance of vector-derived factors that includes sand fly saliva ^40^ and microbiota ^29^, and parasite by-products such as the promastigote secretory gel ^41^, exosomes ^42^ and other virulence factors ^43^, that have the potential to modify and impact vaccine efficacy ^44^.

A proviso for a vector transmitted leishmaniasis CHIM is to have an efficient protocol to allow sand flies to safely bite human volunteers. Previous studies ^45– 47^ have demonstrated that controlled sand fly biting on humans is achievable and a protocol to examine human immune responses following sand fly bite has recently been developed ^48^. The specific aims of the current study are to assess the biting frequency and proportion of *P. papatasi* and *P. duboscqi* females successfully taking a blood meal when exposed to human skin and to record adverse events through clinical examination including dermatoscopy, evaluation of routine biochemistry and the use of a visual analogue scale assessing severity of signs and symptoms. In addition, following a previous public involvement (PI) consultation group exercise ^49^, that was used to shape the current protocol, we conducted a follow-up focus group with volunteers to record their attitudes and experiences of the FLYBITE study, and to help refine the development of a cutaneous leishmaniasis CHIM. This study is reported in line with the Consolidated Standards of Reporting Trials (CONSORT) guidelines ^50^.

## Methods

### Ethics statement

The study was approved by the UK National Health Service Research Ethics Committee on the 2 ^nd^ August 2019 (Reference: 19/SC/0297; project ID 266151); and by the Department of Biology Ethics Committee, University of York on the 9 ^th^ September 2019 (CL201908). The study was registered at www.clinicaltrials.gov (NCT03999970) on the 27 ^th^ June 2019 ^51^. 

### Study design

The study was conducted in the Translational Research Facility at the University of York, UK and was a non-randomised, participant-blinded clinical study in healthy participants with two parallel arms and a 1:1 allocation. In total, 12 participants were recruited to two study groups, one group exposed to *P. papatasi* and one exposed to *P. duboscqi*. Sample size was based on a number of factors including the sample size used in similar CHIM studies in the development of new models, for example in the pilot CHIM studies for malaria ^52, 53^. In these models, 4 and 5 participants were recruited respectively. Previous data suggests the take rate for *Leishmania* in a controlled setting is 82.6% ^32^, in comparison to the higher take rate in the more efficient malaria model. Therefore in our model using two sand fly species, we determined that enrolling 6 participants per species was the most appropriate number to balance safety and efficacy. Recruitment was via advertisement within the University of York as well as local media, and both internal and external websites ^54^. The participants were allocated to each group based on availability of sand flies without randomisation. The study was conducted according to the principles of the current revision of the Declaration of Helsinki 2008 and ICH guidelines for GCP (CPMP/ICH/135/95). All participants provided written informed consent for the sand fly biting study and the focus groups prior to enrolment.

Participants were compensated for their time and inconvenience at the following rates in relation to each visit: screening visit, £60; sand fly biting visit, £100; 3 follow-up visits, £25 per visit; focus group, £60.

### Outcome measures

The primary outcome measure was safety and effectiveness of the sand fly biting protocol, with effectiveness being defined as the number of sand fly bites sustained (Extended Data 1 ^50^). Effectiveness was assessed by visualisation of sand fly bites immediately after the biting procedure, participant reported biting sensation and investigator-reported sand fly engorgement. Clinically visible evidence of bites following removal of the sand fly biting chamber were identified using dermoscopy and digital photography and the bites then counted. These counts were verified by two study researchers. A proportion of these images were later checked by study clinicians to ensure accuracy and consistency. Safety was assessed by adverse event data collection through clinical history, clinical examination & blood testing, as well as participant-reported experience from diary card data.

The secondary outcome measures were the recorded response to sand fly bite on human participants, and participant’s attitudes to sand fly biting. Human response was measured by clinical photography and dermoscopy, a routine blood panel including inflammatory markers, total immunoglobulin E (IgE), and the development of antibody responses to sand fly salivary gland proteins. A post-study focus group with study participants was undertaken to determine their experiences of taking part in the research.

### Eligibility criteria

Healthy male and female subjects aged between 18 and 65 years old were eligible. Participants were screened for potential immunodeficiencies based on blood-borne virus testing and full blood count, as well as clinical examination and clinical history. Participants were excluded if they had a past history of leishmaniasis (determined by rK39 *Leishmania* antibody rapid diagnostic test (IT LEISH, Bio-Rad)), had received recent immunizations, immunoglobins or blood products that could interfere with any serological analysis, any history of significant skin conditions, atopy, anaphylaxis or other serious reactions including significantly raised IgE at baseline. IgE was measured given the ease of processing in comparison to serum tryptase, and its relationship with active atopic diseases ^55^, and the subsequent relationship between atopy and anaphylaxis ^56^. The risk of previous *Leishmania* infection undetectable by serology and sand fly exposure was assessed and any participants with recent or prolonged history of travel to regions where *Leishmania*-transmitting sand flies are endemic were excluded. Inclusion criteria included willingness to give consent to refrain from travel to *L. major*-endemic regions during the study.

### Maintenance of sand flies

*P. papatasi* and *P. duboscqi* sand flies were obtained from a colony maintained at Charles University, Prague. The colony is reared based on extensive experience and consensus on sand fly rearing ^57, 58^. Colonies were routinely screened for Phleboviruses and Flaviviruses. Sand flies were transported to a secure insectary at the University of York between days 3 to 5 of reaching the adult stage of development as holometabolous insects. Sand flies were maintained at 26°C and 70% humidity with a photoperiod of 12 hours light and 12 hours dark, within 40cm ^2^ nylon insect cages with a feeding membrane window (BugDorm, MegaView Science Co., Ltd., Taiwan). Sand flies were maintained on a sucrose solution between blood feeding (comprised of cotton wool soaked in a 50% sucrose solution) and starved 18 to 25 hours before a blood meal at age 5 to 7 days. A rabbit blood meal was provided via a membrane (Hemotek membrane feeding system) for up to 1 hour in the dark ^59^ before engorged females were separated. Male sand flies were present during feeding to increase the rate of feeding ^58^.

### Preparation of the biting chamber

On the day of the clinical study, five female sand flies were placed into a custom-built watch-like biting chamber (Precision Plastics Inc, Maryland, USA). This occurred 12 to 15 days post-blood meal, at which point sand flies were aged 18 to 21 days. All pre-biting procedures were conducted on ice to reduce sand fly metabolic activity ^60^.

### Study procedures and intervention

***Pre -screening visit.*** All potential subjects had a pre-screening assessment conducted by either a clinician or study nurse either face-to-face or via telephone to determine eligibility and availability. This included general health status, allergy history, and any history of leishmaniasis infection or prolonged residence in an area where *Leishmania*-transmitting sand flies are endemic.

***Screening visit.*** Screening visits were conducted by clinical study investigators from 30 to 7 days prior to the sand fly biting visit. After written informed consent, the subjects underwent a full medical history and clinical examination. A routine panel of blood samples were provided for haematology, renal and liver function and C-reactive protein (CRP). A blood-borne virus screen (Hepatitis B surface antigen, HIV antibodies, Hepatitis C antibodies), serum β-Human Chorionic Gonadotropin for female participants and an rK39 *Leishmania* antibody rapid diagnostic test (IT LEISH, Bio-Rad) was also carried out. Further blood samples were taken for downstream experimental and exploratory analysis including peripheral blood mononuclear cells (PBMCs) and testing for sero-evolution using an ELISA assay for antibodies to sand fly salivary proteins as described elsewhere ^61, 62^.

***Sand fly biting visit.*** Up to two participants underwent sand fly biting on any given day, and no participants underwent sand fly biting simultaneously. Participants were provided with a neutral, non-scented skin wash to minimise any host factors that could account for variation in inter-participant sand fly biting behaviour ^63– 65^. Either *P. papatasi* or *P. duboscqi* were used depending on availability on the day of sand fly biting. The biting chamber with sand flies enclosed within was placed on the volar aspect of the non-dominant proximal forearm, approximately 2 to 3 centimetres distal to the antecubital fossa. The sand fly biting chambers were secured on the participants arm for 30 minutes. The subjective experience of each participant to biting was recorded including biting sensation, pain and pruritis. A clinician and a research nurse observed the participant during this period.

Although there is no clear consensus or guidelines on the emergency provisions needed during such a clinical study, after review of the literature the study investigators ensured that there was availability of appropriate resuscitation equipment (including defibrillator and medical grade oxygen, and intramuscular adrenaline (1:1000) ^66^ for administration in case of anaphylaxis to sand fly bite).

Non-identifiable video and photography were recorded after written participant consent to document sand fly biting behaviour and any demonstrable evidence of sand fly bites. The participant was observed for an additional 2 hours for signs of any reactions or medical issues following removal of the sand fly biting chamber. Evidence of any sand fly bites present on the skin was recorded by digital dermatoscopy (MoleScope II – Mobile Dermatoscope attached to an Apple iPhone 7). Study investigators examined the sand flies for evidence of blood-feeding by presence of a red translucent swollen abdomen on visual and microscopic inspection. Participants were given a diary card to record daily features at the sand fly bite site and any systemic adverse events. In each vector intervention arm, there was a further 1:1 allocation of the biting aperture size to either 6mm or 8mm.

***Follow-up visits.*** Follow-up visits took place at day 3 (±1 day), 10 (± 3 days) and 21 (±5 days) following the biting visit. Participants were assessed for local and systemic adverse events using a focused history, clinical examination, dermoscopy and photography. Repeat blood sampling for a routine panel, PBMCs and serum were taken at each follow-up visit. Urinary *β*-human chorionic gonadotropin was tested in women at the day 10 and 21 visits. Serum IgE was taken at the day 3 and 21 visit. Serum for ELISA assay for sand fly salivary gland protein antibodies was taken at the day 21 visit.

***Focus groups.*** Focus group discussions at the end of the clinical study (after the last participant’s final follow-up visit) assessed the participants’ experiences in-depth including acceptability, to inform the design of the subsequent CHIM study. Two focus group sessions took place with five participants from the sand fly biting study in each group (total n=10). The participant numbers were compatible with accepted methodology for focus groups ^67, 68^. The focus groups were recorded using digital audio with consent and each session lasted approximately 3 hours. The proceedings took place in accordance with a pre-prepared schedule, with specific questions agreed in advance with all investigators, to ensure appropriate coverage of key topics (Extended Data 2 ^50^). Both sessions were chaired by GJ who acted as the independent facilitator and field notes were taken. The dialogue was fully transcribed verbatim and analysed using thematic analysis ^69^, assisted by NVivo Pro software (version 12 QSR International Pty Ltd). Transcripts were not returned to participants. Data collection used a mixture of prescribed questions from the focus group schedule as well as open-ended discussion in order to achieve data saturation. Alternative open source software for qualitative analysis are available such as ATLAS.ti ^70^.

Thematic analysis is a flexible approach for engaging with, identifying and analysing the meanings inherent within qualitative data. The goal of the analysis was to summarise and interpret the participants’ experiences. Therefore, an inductive thematic approach, utilising Braun and Clarke’s six key stages was undertaken ^69^ to identify and prioritise their key concerns and generate relevant themes ^71^. The data was transcribed by a colleague external to the research team. Several members of the team independently listened to the audio-recordings and read the two focus group transcripts (VP, GJ, NM). To establish the trustworthiness of the analysis, coding, at a semantic level, was undertaken to label items of interest in the data (NM) ^68^. A second member of the team independently checked a proportion of coding against the transcripts (GJ). Together, and in an iterative process, NM and GJ actively searched and reviewed where codes clustered together to generate the key themes within each and across both focus groups.

Discussion of, and agreement upon, common patterns and broader themes from the participants’ experiences was reached by consensus. Any discrepant views and areas of diversity were considered and discussed with the wider study team. Clinical investigators were present to discuss the results of the study and to discuss future CHIM studies but were not present during discussion on attitudes to participation in the study.

### Data analysis

Data tables are reported as mean (SD) or n (%). Groups were compared using independent t-test (continuous data), Mann Whitney (ordinal data) or chi-square tests (categorical data). A p-value of <0.05 was considered to indicate statistical significance. All analyses were performed on IBM SPSS Statistics for Windows (Version 26.0. Armonk, NY; IBM Corp). Summaries of adverse events reported in the study are presented as summed data for all participants per adverse event or summed VAS score for each participant across all adverse events. Data are presented as median and range. Graphs were generated in GraphPad Prism v9.0.1. Alternate statistical packages could be used.

## Results

### Participant characteristics

**Figure 1.**
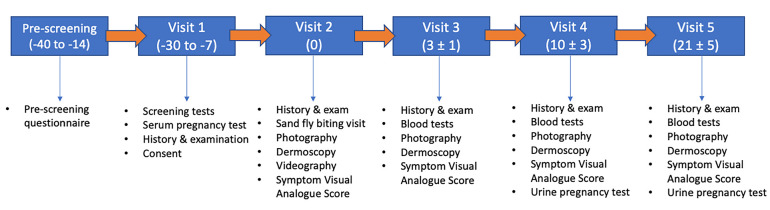
Schedule of events. Figure illustrates schedule of events with day of visit given in relation to biting visit (Day 0). Window for visits is indicated in brackets.

**Figure 2.**
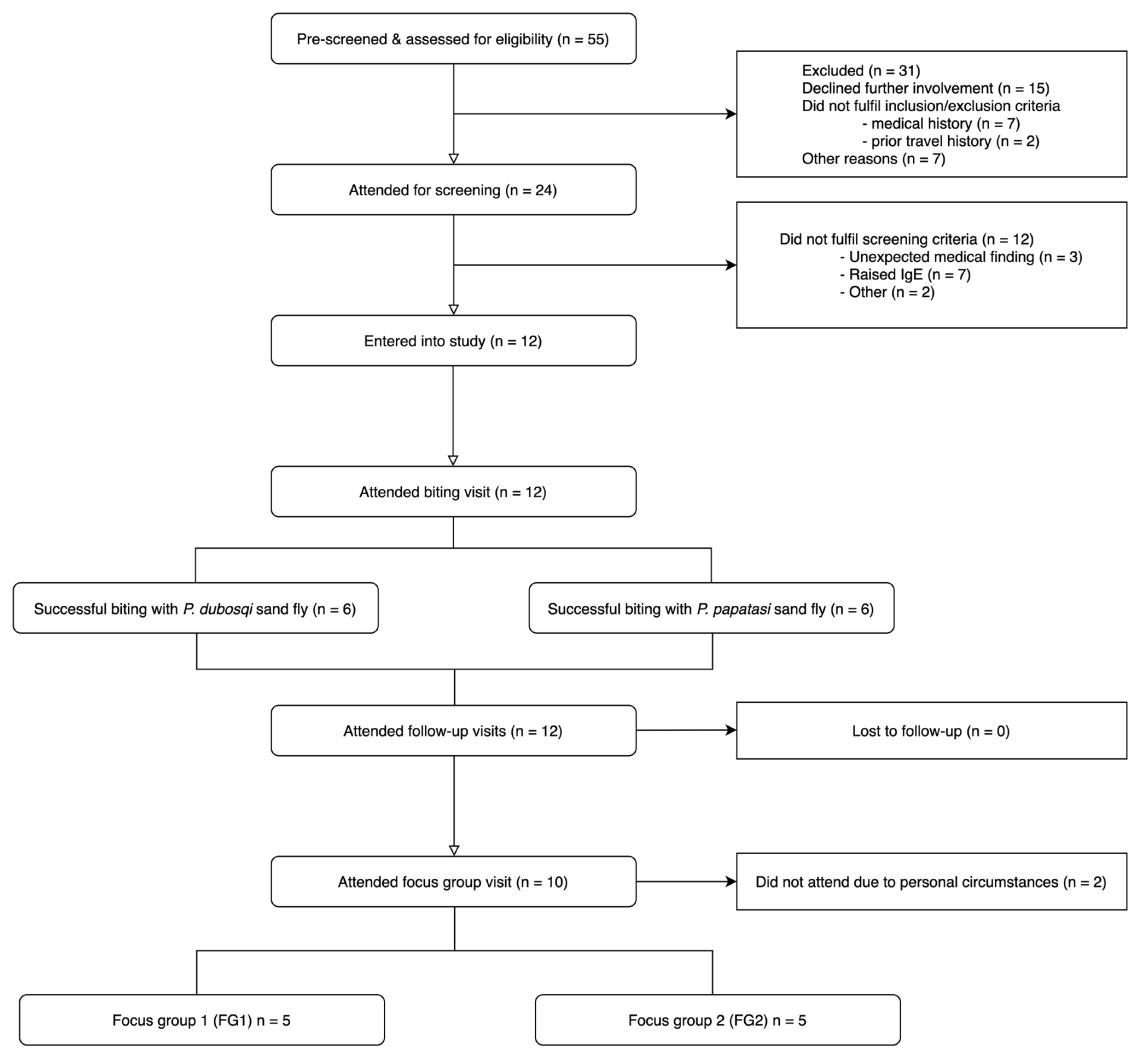
CONSORT (Consolidated Standards of Reporting Trials) flow diagram.

Recruitment and screening took place from October to November 2019 and the follow-up period lasted until January 2020 ( Figure 1 and Figure 2; Extended Data 3). 55 participants were pre-screened and assessed for eligibility, of which 24 attended for screening. In total, 12 participants were deemed eligible for entry into the study and were allocated to the *P. papatasi* arm (n = 6) or the *P. duboscqi* (n = 6) arm based on availability of sand fly species and date of recruitment. 100% (12/12) of participants completed study visits to day 21 post-sand fly biting and none were lost to follow-up. 10/12 attended for the focus group exercise. Two participants were unable to attend the focus groups due to unforeseen personal circumstances unrelated to the study. The majority of exclusions following screening were due to raised serum IgE levels. Participant demographics and clinical characteristics at baseline are provided ( Table 1; Extended Data 4 and 5 ^50^). Gender representation was similar in each arm, with five female participants and one male participant recruited to each arm. Mean age in the *P. papatasi* arm was 40.8 years±12.8 years, compared to the 39.5±11.9 years in the *P. duboscqi* arm. Participants had no unusual skin conditions; one had a tattoo and one a previous scar from an insect bite.

**Table 1.** Baseline participant characteristics.

		Sand fly species			
		*Phlebotomus* *duboscqi*		*Phlebotomus* *papatasi*	
		n	%	n	%
Gender	Female	5	83%	5	83%
	Male	1	17%	1	17%
Eczema	No	4	67%	5	83%
	Yes	2	33%	1	17%
Asthma	No	6	100%	4	67%
	Yes	0	0%	2	33%
Urticaria	No	6	100%	6	100%
Psoriasis	No	6	100%	6	100%
Self-reported propensity to scarring	No	6	100%	6	100%
Allergy to any medications (including over- the-counter items)	No	4	67%	4	67%
	Yes	2	33%	2	33%
Allergy to non-medications (including hay fever)	No	4	67%	4	67%
	Yes	2	33%	2	33%
History of anaphylaxis	No	6	100%	6	100%
Smoking history	Current	0	0%	1	17%
	Never	5	83%	3	50%
	Former	1	16.70%	2	33%
Travel outside the UK in the last 12 months	No	1	16.70%	2	33%
	Yes	5	83.30%	4	67%
Travel outside Europe in the last 12 months	No	6	100.00%	4	67%
	Yes	0	0.00%	2	33%

### Primary outcome measures

**Figure 3.**
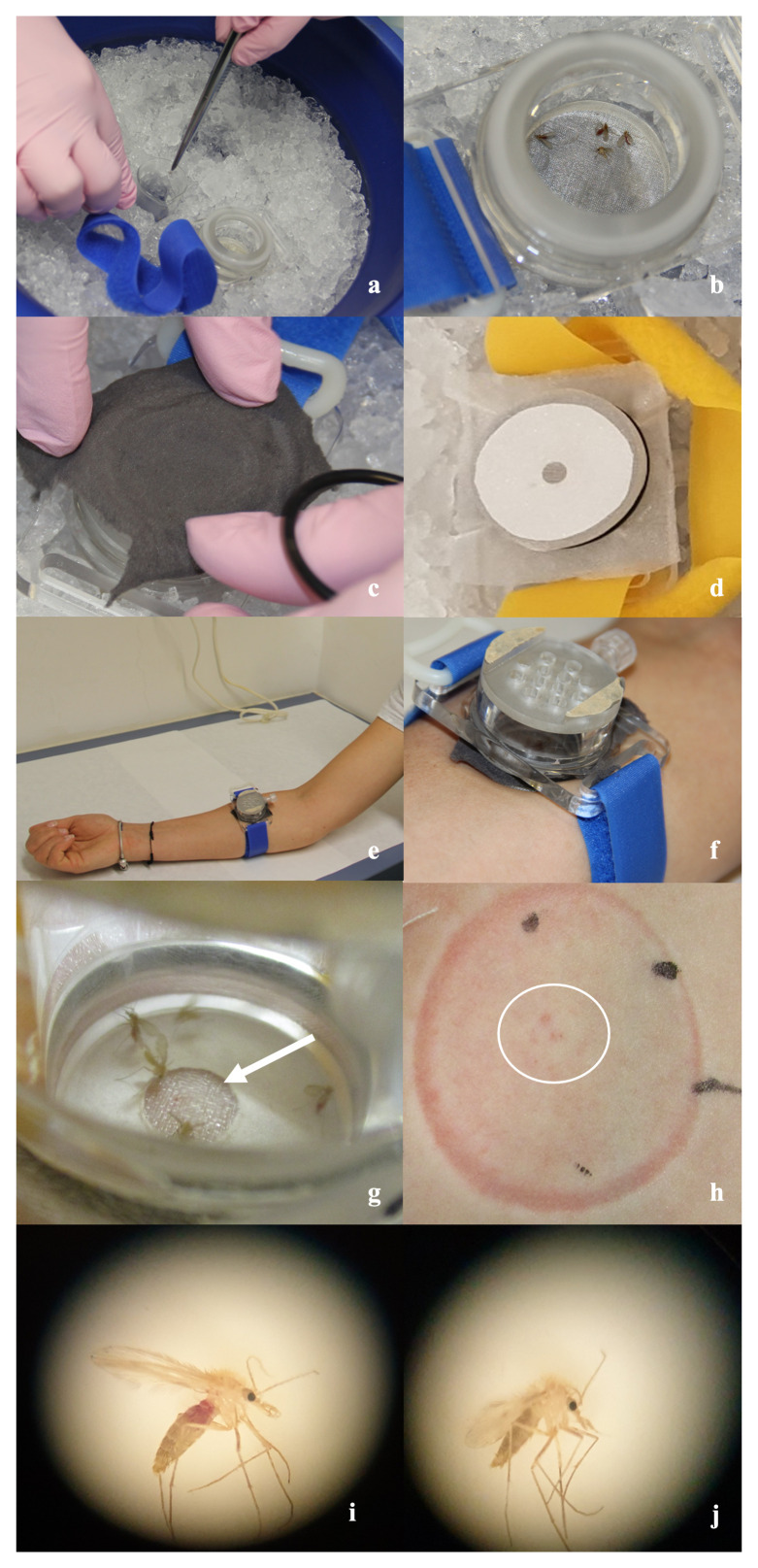
Sand fly biting chamber and procedures. Photographs to illustrate key steps in the sand fly biting procedure. ( **A** & **B**) Using fine tweezers, 5 sand flies are placed inside the biting chamber on ice. The sand fly biting chamber is approximately 5cm in diameter. ( **C**) A gauze covering is placed over the bottom of the biting chamber with sand flies positioned inside. ( **D**) Filter paper is used to form an aperture of between 6-8mm to limit the area of sand fly biting. ( **E** & **F**) An adjustable Velcro strap is used to customise the fit for each participant, and biting chamber placed 3–4cm distal to the antecubital fossa. ( **G**) Sand flies within the biting chamber; biting aperture with gauze visible (arrow). ( **H**) Participant skin demonstrating pressure mark from biting chamber and small visible bite marks (circled). ( **I**) Microscope image of sand fly, following biting on participants, with red swollen abdomen demonstrating blood meal has been taken and ( **J**) sand fly following biting on participants with absence of red swollen abdomen suggesting blood meal has not been taken.

Participants were exposed to sand flies in a bespoke chamber for 30 minutes ( Figure 3; Extended Data 6 ^50^). 100% (12/12) of participants received at least one successful sand fly bite. There was no apparent difference between sand fly species in terms of mean number of sand fly bites (7.00±2.76 vs 6.33±5.39 for *P. papatasi* and *P. duboscqi,* respectively). Comparison of sand fly biting rate, defined as number of bites per 5 sand flies in 30 minutes also showed no significant difference (3.33±0.81 vs 3.00±1.26 bites for *P. papatasi* and *P. duboscqi,* respectively) although the study was not powered to detect a significant difference ( Table 2). Successful biting was confirmed by the demonstration of red translucent swollen sand fly abdomens on dissection subsequent to removal of the chamber. In all cases at least one sand fly demonstrated these characteristics for each participant. There was no significant difference in the number of engorged sand flies post-biting between species (3.33±0.82 vs 3.00±1.27 for *P. papatasi* and *P. duboscqi*, respectively) ( Table 3). Bites remained visible at the final visit at day 21, although they had reduced in number (3.17±1.60 vs 3.50±3.73 for *P. papatasi* and *P. duboscqi*, respectively). Hence, the protocol was deemed to be 100% effective as each participant exposed to 5 sand flies received at least one bite, with at least one sand fly demonstrating evidence of feeding in each case.

**Table 2.** Sand fly biting comparison; Number of bites and biting rate (bites per 5 sand flies).

	Type						p-value
	*Phlebotomus duboscqi*			*Phlebotomus papatasi*			
	Mean	SD	n	Mean	SD	n	
30 mins -Reviewer 1: Number of bites	6	5	6	7	3	6	0.485
30 mins -Reviewer 1: Biting rate	1.3	1.1	6	1.4	0.6	6	0.485
30 mins -Reviewer 2: Number of bites	6	5	6	7	2	6	0.485
30 mins -Reviewer 2: Biting rate	1.3	1.1	6	1.4	0.5	6	0.485
90 mins -Reviewer 1: Number of bites	5	3	6	6	2	6	0.589
90 mins -Reviewer 1: Biting rate	1	0.6	6	1.2	0.5	6	0.589
90 mins -Reviewer 2: Number of bites	5	3	6	6	2	6	0.589
90 mins -Reviewer 2: Biting rate	1	0.6	6	1.2	0.5	6	0.589

**Table 3.** Summary of participant blood testing and bite site examination (at baseline and during follow-up).

		Mean *P.papatasi*	Standard deviation *P.papatasi*	Mean *P.duboscqi*	Standard deviation *P.duboscqi*
Baseline bloods	Total white cell count (x 10 ^9^/L)	6.78	1.61	6.55	2.06
	Eosinophils (x 10 ^9^/L)	0.13	0.08	0.10	0.09
	C-reactive protein (mg/L)	2.67	2.08	1.67	1.15
	IgE (KU/L)	24.53	13.88	38.87	28.13
Biting Day (Day 0)	Flies fed	3.33	0.82	3.00	1.26
	Bites visible (30 minutes)	7.00	2.76	6.33	5.39
	Bites visible (90 minutes)	6.17	2.40	5.00	2.97
Day 3 post-biting	Bites visible	2.67	0.82	3.00	2.97
	Size of biggest lesion (mm)	3.67	2.88	1.00	0.89
	Total white cell count (x 10 ^9^/L)	7.25	2.45	6.38	1.61
	Eosinophils (x 10 ^9^/L)	0.12	0.08	0.13	0.10
	C-reactive protein (mg/L)	5.00	4.24	1.67	1.15
	IgE (KU/L)	22.70	13.44	32.80	23.39
Day 10 post-biting	Bites visible	2.67	1.37	2.83	3.37
	Size of biggest lesion (mm)	3.50	3.56	3.00	4.69
	Participant-reported pain (0-10 VAS)	0.00	0.00	0.00	0.00
	Participant reported itch (0-10 VAS)	0.00	0.00	1.00	2.45
	Erythema	1.00	0.63	0.83	0.98
	Swelling	0.17	0.41	0.33	0.52
	Blistering	0.17	0.41	0.17	0.41
	Total white cell count (x 10 ^9^/L)	7.88	1.43	5.98	1.23
	Eosinophils (x 10 ^9^/L)	0.15	0.10	0.17	0.12
	C-reactive protein (mg/L)	3.67	2.31	7.67	10.69
Day 21 post-biting	Bites visible	3.17	1.60	3.50	3.73
	Size of biggest lesion (mm)	2.50	1.38	2.33	2.42
	Erythema	1.17	0.75	1.00	0.89
	Swelling	0.33	0.52	0.33	0.52
	Blistering	0.00	0.00	0.00	0.00
	Participant-reported pain (0-10 VAS)	0.00	0.00	0.00	0.00
	Participant reported itch (0-10 VAS)	0.00	0.00	0.17	0.41
	Total white cell count (x 10 ^9^/L)	7.47	2.37	6.48	0.69
	Eosinophils (x 10 ^9^/L)	0.10	0.09	0.17	0.14
	C-reactive protein (mg/L)	5.00	1.73	2.00	1.73
	IgE (KU/L)	22.77	12.99	33.13	22.69

### Safety of sand fly bite

Solicited, or expected adverse events included bite site-related itch, pain, erythema, swelling, blistering or bullae, and ulceration. These events were graded based using a modified version of grading systems by the National Institutes of Health (NIH) ^72^ (Extended Data 7 ^50^). Four grade 2 adverse events were noted. One participant was noted to have a new cardiac murmur at screening, prior to sand fly exposure, and this was recorded as a grade 2 adverse event. A further participant had a new cough at day 21, which was clinically suspected to be an unrelated upper respiratory tract infection. Just two study-related grade 2 adverse events were noted, both were solicited: erythema at the bite site and persistent itch at the bite site. No grade 3 or higher adverse events were reported. No suspected unexpected serious adverse reactions (SUSARs) were reported (Extended Data 7 and 8 ^50^). IgE was not affected by exposure to sand fly bite. Mean CRP (normal range <5mg/L) on day 3 post-biting (5.00±4.24 vs 1.67±1.15), day 10 post-biting (3.67±2.31 vs 7.67±10.69) and day 21 post-biting (5.00±1.73 vs 2.00±1.73) was not influenced by species of sand fly ( *P. papatasi* and *P. duboscqi* described respectively). Hence, bite by uninfected *P. papatasi* and *P. duboscqi* is shown to be safe for healthy volunteers.

### Secondary outcome measures: VAS

**Figure 4.**
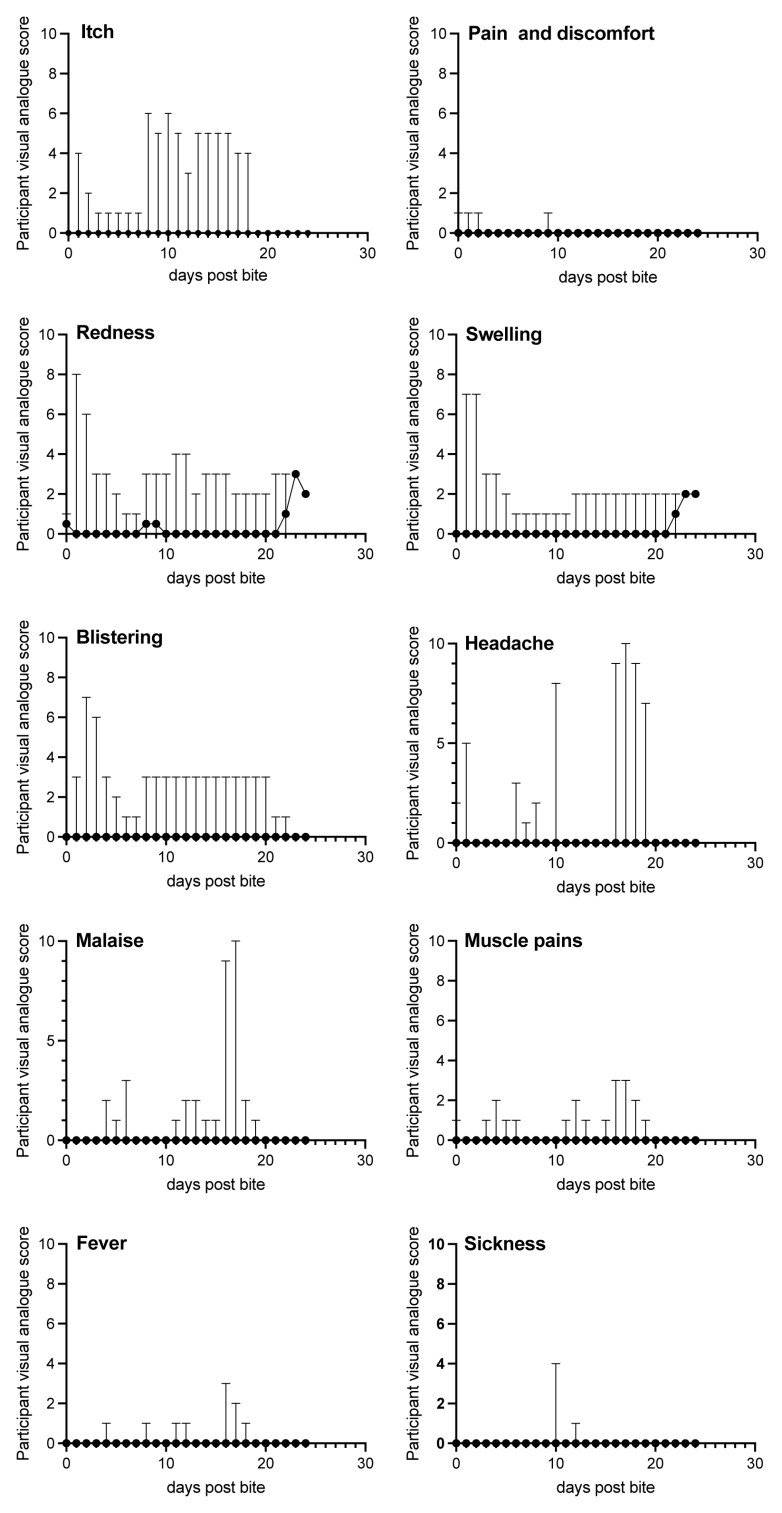
Summed adverse events reported by type during FLYBITE. Adverse events were recorded by each participant at each visit on a visual analogue scale of 0-10 (see Methods). Pooled data for all 12 participants are presented separately for each adverse event (as indicated in panels) at each time point. Data are shown as median (circles) and range (vertical bar).

**Figure 5.**
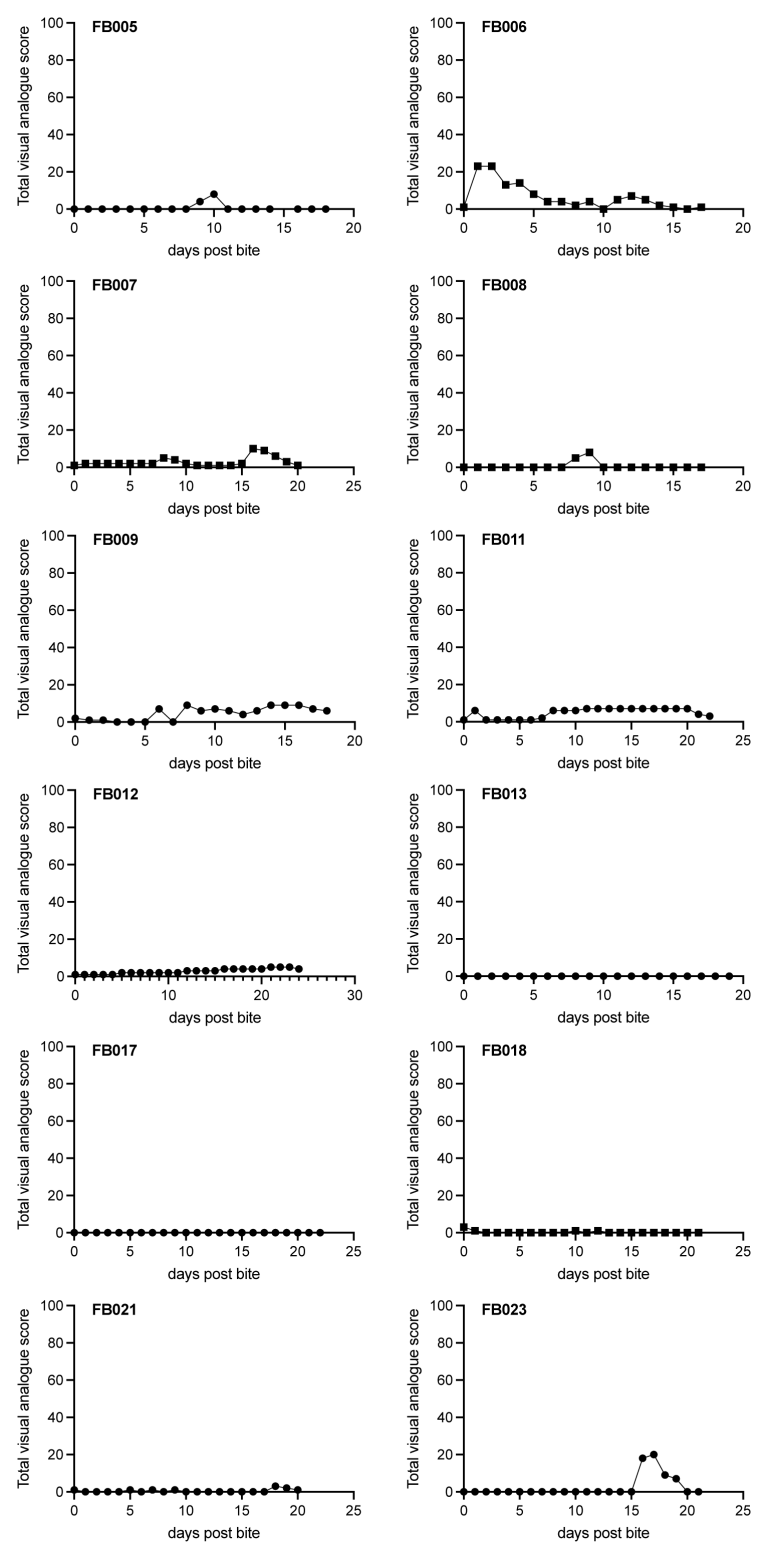
Summed adverse events reported by individual participants during FLYBITE. Ten adverse events were recorded by each participant at each visit on a visual analogue scale of 0–10 (see Methods). Data are presented as the sum of all scores (out of 100) for each participant (as indicated in panels) at each time point.

Participant experience was recorded in both a quantitative (visual analogue daily diary card) and a qualitative manner (post-sand fly focus group). A daily diary card was recorded by all participants from 90 minutes following biting, until the last scheduled visit. There was a 100% response rate. A visual analogue scale (VAS ^73^) on a 10 point scale was used by participants to record their daily experience for each of the following: itch, pain/discomfort, erythema, swelling and blistering at the bite site ( Figure 4 and Figure 5; Extended Data 9 ^50^). Systemic effects such as headache, malaise, myalgia and fever were also recorded. The mean VAS scoring was between 0–1 for each measure until day 21. The most commonly reported effects were localised erythema, swelling and itch. Based on this diary card data, the sand fly biting was well-tolerated with minimal adverse effects. Two participants reported an intercurrent viral upper respiratory tract infection, which may account for the increased incidence of malaise, myalgia and headache close to the end of the recorded diary card data. The viral infection was self-limiting in both cases and had resolved by the final visits. There was some recrudescence of swelling as reported by some participants towards the end of their study involvement, although this was minimal ( Figure 5).

Some participants attended after the day 21 visit and were allowed to record diary card data until this visit. The mean score for erythema and swelling for these days was between 2–3, although there were only limited participant responses on these days, as most participants had completed their involvement in the study at this point. Additionally, the window period for each particular visit ( Figure 1) also resulted in some participants attending for their final visit at day 18 post-biting.

### Focus groups

In total, 10 participants took part in two focus groups (FG1 and FG2). Each focus group consisted of five participants (each had four female and one male participant). In FG1, the age range of the females was 26 –51 years; in FG2, the age range was 21–59 years. The male participants were aged 38 and 40 years old, respectively.

Overall, four overarching themes were identified: i) recruitment and quality of the participant-facing information, ii) screening process, iii) experience of being bitten, and iv) overall study experience. These themes are also described with the sub-themes and exemplar quotes (Extended Data 10 ^50^). The similarity in themes across the two focus groups is demonstrated within a coded comparison diagram (Extended Data 11 ^50^). In particular, positive themes concerning the biting aspect, and the lack of significant post-bite reaction was shared by both focus groups. Full transcripts of the focus groups are provided (Extended Data 12 & 13 ^50^).

### Recruitment and quality of the patient-facing information

The participants had become aware of the study via a number of recruitment methods including local and national advertisements in posters (Extended Data 3 ^50^), newsletters and mailing lists. Whilst the study website was perceived as clear and informative, it was not widely accessed by the participants prior to their involvement. The main reason cited for not using the website was because the investigators had provided all the information the participants required during initial contact, although it was suggested that the website should play a bigger role in future studies. Participants suggested verbal methods including talks and presentations might attract a wider demographic and help overcome information fatigue. Word-of-mouth was also reported to have played an important role in recruitment. With respect to clarity of message some participants commented on the importance of stating there would be financial compensation for taking part within the text of the advertisement although opposed specifying the exact amount. It was also commented that the use of social media would be beneficial in future advertising campaigns. The inclusion of a participant wearing a sand fly biting chamber on the poster was welcomed, although it was felt that there should be an effort to make the overall aesthetic more visually appealing in future.

*“what appealed to me about the poster was that you actually showed the biting chamber because I think that was really useful because otherwise, I would have thought that I’d have perhaps been in a room being bitten by flies” (P10, FG1)*

Participants stated that although the written material clearly specified the methods and details of the project, there should be greater focus on the humanitarian nature of the project and perceived end-goals. The quality of the participant-facing written information was well received although some felt that more information about the medical examination was needed.

### Screening process

The participants found the screening process beneficial in developing understanding of the project, exposure to scientific researchers and for generalised health screening. Indeed, some of the participants volunteered that the process had uncovered a number of underlying health conditions for which they were now able to seek medical attention. It was felt that the screening process and testing had helped the participants to build a relationship with the team, and the continuity of care of the clinical team strengthened these positive relationships. Some participants described displeasure at the routine blood testing in general, including some pain and technical difficulties obtaining venous access. There was also some anxiety associated with waiting for pregnancy test results. Participants did however express gratitude for the testing in general. For some, the screening turned out to be more extensive than they had initially expected, although they were pleased overall to have undergone a thorough examination.

*“I thought it was quite nice. I know I’m healthy. I just didn’t realise that was going to be as extensive as it was.” (P6, FG1)*

### Experience of being bitten

The participants’ experiences of being bitten by sand flies was overwhelmingly positive. The sand flies were smaller than had been anticipated and the biting chamber was deemed to be relatively innocuous.

*P14: “They were quite small yeah. I was expecting a bit bigger. (FG2)*

*P15: Because you couldn’t really see them.” (FG2)*

Overall, the participants felt that taking part in the study had been *‘unremarkable’*, with no unexpected issues during the sand fly biting and events taking place as in line with prior explanation. The local cutaneous effects of the sand fly bite were minimal and the skin reaction generally had been less than had been expected. Overall, the bite was not painful but some participants experienced some minor blistering shortly after.

*P7: “It [the bite] was unremarkable to the point where I forgot I was taking part in a medical study.” (FG1)*

One participant had skin itchiness and whilst they needed to take over-the-counter antihistamines no other medication was required by any of the other participants. Another felt “glad” they had a small reaction as it demonstrated something was “happening”, and others described their curiosity and even *‘excitement’* when the flies started to bite and witnessing them engorge.

*P12: “[The bite itself] was really minor, much less, I mean just a tiny red mark and I was expecting you know a kind of a horrible itchy lump so it was much less than I expected.” (FG1)*

*P15: “[The bite] was just itchy so the more I itched it and then it got the skin slightly torn and then just looked like an insect bite, just a scratched insect bite that I would itch which made it worse.” (FG2)*

However, there was some pre-bite anxiety. The participants described *‘worrying’* that biting might hurt, and *‘feeling slightly anxious’*. But whilst the process was uneventful for most, it took longer than anticipated and the environment was felt to be clinical and a little uncomfortable during the waiting period post-bite. The blood sampling procedure was perceived to be more uncomfortable than the sand fly biting itself. Some participants struggled to remember to complete the post-bite diary. For some, a paper-based diary was acceptable but others suggested that a phone app or text reminders on their phone might be useful. With the exception of one participant who struggled to remember the advice they were given, the remaining participants felt confident they had received all the information they needed to get in touch with the study team. They also described positively the follow-up support networks that the team had put in place.

### Overall study experience

Overall, the participants described their experiences in the study positively and felt well-informed throughout. In particular, they described the team as professional and providing good communication.

*P7: “it [involvement with the study and study team] was the most professional thing that’s happened to me in a long time.” (FG1)*

The participants also enjoyed the social interaction with the study team and felt they had developed a good relationship with them, with one participant describing the team as *‘warm’*. The participants also felt they were given enough opportunity to ask questions, had no safety concerns and felt appreciated, valued and well-cared for throughout the whole study.

***Investigator: “Did you have any safety concerns at all when you were part of the project? The trial.***

*P(Several): No. (FG1)*

***Investigator: None at all?***

*P(Several): No.” (FG1)*

At no point did any of the participants want to exit the study. However, for some, the time commitment was more than they had anticipated. Also, whilst they acknowledged the time commitment and would be willing to use other virtual or electronic follow-up methods, they still felt in-person contact was important and would wish this to continue. 

*P16: “Well I work better with paper. I’m still a dinosaur when it comes to all the sort of technology and everything so for me it was fine to have to you know didn’t forget and took it with me and everything so yeah.” (FG2)*

Finally, most of the participants thought the remuneration for taking part in the study was satisfactory and appropriate for the time commitment it necessitated. Despite this, the participants expressed differences of opinion regarding the importance of remuneration, with some stating that this was important versus others stating that it was not relevant. Most of the participants expressed that they would have taken part in the current study for less and that the money was a bonus and secondary to their interest in the study. Although for one participant the financial incentive was the key motivator in their decision to take part (albeit they donated the money to charity).

*P3: “getting some compensation makes you feel like your time is valuable, you know I have very little free time and to do it for nothing and take a lot of time off I would be reluctant to do that […] I don’t think everyone should have to do it for free, if you’d like to it’s not a bad thing to get a bit of money as part of it.” (FG1)*

 Other reasons given were a genuine altruistic interest in the condition/research, a belief that the study was meaningful and worthwhile, and a desire to help others in the advancement of science and world health. They felt these aspects of the study should be incorporated into any future recruitment and participant-facing materials in the future CHIM.

*P16: “it’s my chance to sort of contribute to some sort of research which in the long run is going to hopefully benefit quite a few people.” (FG2)*

## Discussion

Deliberate human transmission of *Leishmania* has been used for centuries as part of cultural vaccination practice, typically using a sharp object to introduce the parasite ^33^. Contemporary studies, after discovery of improved parasite culture techniques, have also demonstrated infection by introduction of *Leishmania* parasite to human subjects for both cutaneous and visceral forms ^32, 74– 76^. Once the phlebotomine sand fly vector was implicated in transmission, it was demonstrated to be the natural vector through indirect but deliberate human infection ^77^.

Sand fly biting studies have a long history, although the majority of studies have involved animal models. However, human exposure to non-infected sand fly bite has been studied both in order to attempt transmission to sand flies from a cutaneous lesion (xenodiagnosis) ^78^, as well as demonstration of the sand fly bite reaction ^47, 79^. Several studies have since demonstrated transmission of *Leishmania* to human subjects using phlebotomine sand flies ^80– 82^. A single individual was exposed to the bite of *L. arabica*-infected *P. papatasi* as part of a study on cross-protection, though no lesion developed ^83^. We have described elsewhere a new *Leishmania major* strain suitable for use in a CHIM, with production of a clinical bank at Good Manufacturing Practices (GMP) and validation of its ability to infect *P. papatasi* and *P. duboscqi* sand flies and be transmitted to rodents through sand fly bite ^84^.

The natural sand fly vectors of *Leishmania major, P. papatasi* and *P. duboscqi*, have a similar mode of feeding, and to-date there have been no major studies comparing biting rates on human subjects. This study serves to evaluate the biting rates and reproducibility between these sand fly species in order to determine effectiveness of the study protocol **(Extended Data 1** ^50^ **)**, whilst examining the safety aspects of sand fly biting on humans. There are no reported cases of serious adverse events such as anaphylaxis from exposure to these major sand fly species, although severe reactions including anaphylaxis have been reported rarely in some biting and hematophagous insects ^79, 85, 86^.

It is increasingly recognised that public involvement in research is critical in ensuring high quality outcomes and robust practices and accountability ^87, 88^. As such, prior to undertaking this study we carried out a public involvement (PI) exercise to inform the design and practical considerations in this research area ^49^. The outcomes from this exercise suggested that such a study was acceptable to participants and reinforced the need for clear and thorough written materials in order for true informed consent to be taken. We also describe here a further consultation exercise in the form of a focus group undertaken with the majority of study participants, to understand areas for improvement and barriers to involvement in a future CHIM study for cutaneous leishmaniasis.

All participants received at least one sand fly bite, and our results were in keeping with the known biting studies described. The number of bites sustained by an individual participant was not a factor that we were able to control given the technical and biological factors in animal studies. The number of bites observed by investigators after the removal of the biting chamber was, in the majority of cases, higher than the number of sand flies that fed, due to sand fly probing behaviour prior to settling for a sustained feed ^59^. Of the many well described scoring systems for patient use in dermatological diseases, VAS is used frequently and has been shown to be valid and reliable for assessing dermatological disease in comparison with other scoring mechanisms ^73, 89^. Furthermore, there is a good correlation between VAS used in skin disease, and quality of life measures ^90^. Our VAS data here demonstrates the first known description of a dermatological scoring system used in a sand fly biting study, although this has been described in other arthropod biting studies ^91^ which further strengthens its use with sand fly bites. This study therefore demonstrates the safety and effectiveness of the protocol in preparation for its use in a *Leishmania major* CHIM. 

Given the perceptions that the public may have about deliberate human exposure within studies, public engagement is an important foundation for such projects. The chequered history of such studies add to this viewpoint ^19^. Public involvement in CHIM studies has been well-described in other vector-borne CHIM studies, namely malaria ^92, 93^. With increasing descriptions of new CHIM studies being mooted, so too the need for robust frameworks to ensure appropriate engagement is strengthened. This is especially true of CHIMs for development in LMICs ^94^. The bioethical discussion surrounding such studies has included the utility and practicality of public involvement and engagement within a rapidly changing landscape ^27, 95^. We initially conducted a PI study to inform the development of the FLYBITE protocol and here we report a comprehensive summary of focus group engagement following completion of our study. The key findings from our focus group demonstrate acceptability of sand fly biting on human participants, with routine blood tests, as a benchmark, seen to be less tolerable than the biting itself. Our results are in keeping with those reported by others in arthropod-based CHIM studies, who suggest that the anticipation of harm from such studies fluctuates over time. In our focus group study and the underpinning PI study, it was noted that education was important to overcoming barriers and negative public perception, in common with themes described elsewhere ^92^.

Although this study was successful, there are some limitations. First, it is known that sand fly behaviour is altered following infection with *Leishmania* parasites, although usually this is associated with increased bite rate ^96^. Though previous studies on mice revealed that *L. major*-infected females of both species readily take a bloodmeal on mice ^84^, and *P. duboscqi* infected with *L. major* can cause cutaneous leishmaniasis in mice ^97^ and non-human primates ^98^, we cannot be certain that the biting characteristics on human subjects will be faithfully reproduced using infected sand flies in a CHIM study. The second issue relates to uniformity between participants, which may impact on outcomes from a future CHIM. For example, some participants were noted to have persistent but improving sand fly bite reaction at the final 21-day follow-up visit, whilst most participants had completely healed skin at the site of biting. An extended follow-up period would have allowed for greater characterisation of this variation in response. As *Leishmania major* is associated with lesion development at around 4 weeks post-inoculation, and based on our observations of skin appearance at 21 days post-bites, it may prove difficult to visually distinguish an early cutaneous leishmaniasis lesion from a persistent sand fly bite reaction ^32, 99^. The third issue related to the focus group study, which although was an efficient process and stimulated group interaction that uncovered themes that may not have come to light with individual interviews ^100^, individual interviews have been shown to provide greater understanding of participant knowledge ^101^.

In conclusion, the successful completion of this study complements further research aimed at developing a CHIM model for leishmaniasis. As reported elsewhere ^84^, using a new fully characterised strain of *Leishmania major,* we have manufactured a clinical parasite bank under GMP conditions and confirmed that this isolate is fully transmissible to rodents via the bite of either *P. papatasi* and *P. dubosqi*. How well these preclinical and clinical studies translate into an effective CHIM will be addressed in a subsequent clinical study (LEISH_Challenge; ClinicalTrials.gov ID NCT04512742). If successful, this CHIM will provide a new tool to assess vaccine efficacy allowing subsequent evidence-based decisions to be made on progression of candidate vaccines.

## Data availability

### Underlying data

Open Science Framework: A controlled human infection model for sand fly-transmitted cutaneous leishmaniasis. https://doi.org/10.17605/OSF.IO/H3UCA ^50^.

This project contains the following underlying data:

• Figure 4 Raw data (Summed adverse events reported by type during FLYBITE)
• Figure 5 Raw data (Summed adverse events reported by individual participants during FLYBITE)
• Raw data supporting Extended Data 4 (baseline screening blood tests)
• Raw data supporting Extended Data 4 and 5 (baseline physical examination characteristics)
• Raw data supporting Extended Data 9 (participant-reported diary card data)

### Extended data

Open Science Framework: A controlled human infection model for sand fly-transmitted cutaneous leishmaniasis. https://doi.org/10.17605/OSF.IO/H3UCA ^50^.

This project contains the following extended data:

• Extended Data 1 (Study protocol)
• Extended Data 2 (Focus group schedule)
• Extended Data 3 (Recruitment poster)
• Extended Data 4 (Baseline medical examination characteristics of participants)
• Extended Data 5 (Skin examination findings at baseline)
• Extended Data 6 (Sand fly biting video)
• Extended Data 7 (Adverse event grading system for solicited study events)
• Extended Data 8 (part 1; dermoscopy)
• Extended Data 8 (part 2; dermoscopy)
• Extended Data 9 (Data file supporting Figure 4: Summary of participant-reported diary card data; Visual analogue scoring 0–10)
• Extended Data 10 (Table of themes, with exemplar quotations)
• Extended Data 11 (Coded comparison diagrams of focus group FG1 and focus group FG2)
• Extended Data 12 (Anonymised transcripts for focus group FG1)
• Extended Data 13 (Anonymised transcripts for focus group FG2)
• CONSORT 2010 Checklist

### Reporting guidelines

Open Science Framework: CONSORT checklist for ‘A clinical study to optimise a sand fly biting protocol for use in a controlled human infection model of cutaneous leishmaniasis (the FLYBITE study). https://doi.org/10.17605/OSF.IO/H3UCA ^50^.

Data are available under the terms of the Creative Commons Zero "No rights reserved" data waiver (CC0 1.0 Public domain dedication).

### Consent

Written informed consent for publication of the participants details and their images was obtained from all participants.
