# Supplementary material for: A clinical study to optimise a sand fly biting protocol for use in a controlled human infection model of cutaneous leishmaniasis (the FLYBITE study)
Source: Wellcome Open Res. 2021 Jun 30;6:168. [Version 1] doi: 10.12688/wellcomeopenres.16870.1 (PMC8506224; doi:10.12688/wellcomeopenres.16870.1)
Supplement: Peer review history

# Peer review: Reviewer response for version 1

**DOI:** 10.21956/wellcomeopenres.18609.r46214

**Published:** 2021-10-11

**Authors:** Yoshihisa Hashiguchi

**Affiliations:** 1 Department of Parasitology, Kochi Medical School, Kochi University, Nankoku, Japan

This article on a clinical study to optimize a sand fly biting protocol for use in a controlled human infection model (CHIM) of cutaneous leishmaniasis (CL) through the fly bite study is interesting and important for future steps aimed at vaccine development. The study employed *Leishmania major*, one of the less human pathogenic species in the genus and the natural vectors *Phlebotomus* *papatasi *and *P. duboscqi*, and human participants (volunteers). The results validate a protocol for achieving successful sand fly bites in humans that is safe, well-tolerated and acceptable for study participants. In vector-borne diseases such as leishmaniasis and malaria, an establishment of CHIM would be crucial and provide a novel method for early evaluation of prophylactic efficacy of candidate vaccines.

 Historically, as mentioned by the authors, deliberate human infection with live or attenuated *Leishmania *parasites had been practiced for centuries by people living in the Middle East and former Soviet Union regions, where CL is highly endemic. Build on the knowledge that cure from CL engendered protection against reinfection (“leishmanization”), scrapings from active lesions were used to cause skin lesions at a selected site of the body, thereby avoiding the stigmatization associated with CL scars (overviewed by Mohebali et al., 2019).  Such available information supports the idea or belief that the leishmaniases are vaccine preventable disease, including demonstrable immunity following infection and self-cure; “Leishmanization” in areas endemic for CL involved the inoculation of live virulent (cultured) parasites into cosmetically less conspicuous body areas, typically on the buttock. More defined experimental studies were conducted sporadically through the 20 ^th^ century, evaluating vaccines for leishmaniasis. In recent years, different types of candidate vaccines against leishmaniasis are in development, including an adjuvanted recombinant polyprotein vaccine and RNA-based vaccines. Besides, the authors research group and others reported novel findings regarding the integral nature of sand fly transmission to *Leishmania *infectivity; namely, vaccines inducing protection in mice when infected via needle inoculation (“needle infection”) fail to protect against sand fly-transmitted infection (“fly bite infection”: influence of the saliva).

 The current CHIM tested provides a pathway and an important step for accelerating vaccine development and to more fully understand disease pathogenesis and correlates of protection, including other vector-borne diseases such as malaria and trypanosomiasis, and etc.  Furthermore, as mentioned by the authors, the successful completion of this study complements further research aimed at developing a CHIM model for leishmaniasis, using the authors’ fully characterized strain of *L. major*; the isolate is fully transmissible to rodents via the bite of both *P. papatasi *and *P. dubosqi.* Again, the current CHIM model tested will provide a new tool to assess vaccine efficacy allowing subsequent evidence-based decisions to be made on progression of candidate vaccines.

 The study is well designed and organized, and the manuscript is well written, followed a logical progression. Finally, it is noteworthy that sand flies tested in this study were provided different blood sources at the first and the second meal, the former, rabbit and the latter, human. So, it would be interesting to investigate how those blood-meal differences would influence the outcome of such a CHIM study.

Is the work clearly and accurately presented and does it cite the current literature?

Yes

If applicable, is the statistical analysis and its interpretation appropriate?

Yes

Are all the source data underlying the results available to ensure full reproducibility?

Yes

Is the study design appropriate and is the work technically sound?

Yes

Are the conclusions drawn adequately supported by the results?

Yes

Are sufficient details of methods and analysis provided to allow replication by others?

Yes

Reviewer Expertise:

Reviewer Expertise: Leishmaniasis, Leishmania biology, transmission, and epidemiology.

I confirm that I have read this submission and believe that I have an appropriate level of expertise to confirm that it is of an acceptable scientific standard.

# Peer review: Reviewer response for version 1

**DOI:** 10.21956/wellcomeopenres.18609.r45202

**Published:** 2021-08-23

**Authors:** Paul Bates

**Affiliations:** 1 Division of Biomedical and Life Sciences, Faculty of Health and Medicine, Lancaster University, Lancaster, UK

This article on developing a sand fly biting protocol for use in controlled human infection models (CHIM) of leishmaniasis is an important step towards the goal of testing and ultimately deploying vaccines against the leishmaniases, a complex group of diseases caused by many *Leishmania* species, but which have certain features in common. As noted by the authors the majority of the leishmaniases affect the skin, whilst some species can cause visceral disease, but even the latter originate as skin infections in which what happens at that site certainly influences whether the infection metastasises to visceral sites. Therefore, studies such as this that will enable use of natural transmission via the skin in CHIM are critical. I agree that there is good evidence that the leishmaniases are vaccine-preventable diseases, but would be cautious that they could be eliminated, given that the majority are zoonoses, and even those that are supposedly anthroponotic in transmission have zoonotic roots. Therefore, we will have to live with the threat of leishmaniasis for the foreseeable future, but vaccines have the potential to prevent that translating into clinical human disease.

 The study is well designed, clearly presented and could be replicated by others with access to the biological resources required. Safety is clearly a paramount concern in CHIM, so the choice of *Leishmania major* and its vectors is a wise one that I fully support, this being one of the less pathogenic human species, with no known ability to cause visceral disease. However, it is important to remember that the various parasite species are not the same as each other, with the ability to cause visceral disease (for *L. donovani* and *L. infantum*) or mucocutaneous disease (for *L. braziliensis*) being the most important differences. At some stage the question of translation of results with *L. major* to these other species will become relevant, in other words, how well will vaccine candidates developed against *L. major* work against these more dangerous species? CHIM with these species will probably never been justified on safety grounds, but what this means is that using CHIM that are as faithful to the natural infection process as possible should be an ongoing priority, and this is the case with the work described here.

 As the authors comment we now know there are many important differences between infection by needle inoculation versus infection by sand fly bite. I read the protocol for handling the flies with interest and noted that the bites being used with the human volunteers were those from a second blood meal, the females having been given a first blood meal of rabbit blood from an artificial feeder. Although not directly commented upon, this approach will enable subsequent work to be performed where the first blood meal can be seeded with parasites, thus enabling a challenge with infected sand fly bite that will be in other respects using flies like those described in this study. As mentioned, there are important behavioural differences between uninfected and *Leishmania*-infected sand flies, for example, the latter are much more persistent in their feeding behaviour, but still the approach described here should be valid.

 There did not appear to be any significant differences between the results using *Phlebotomus papatasi* and *P. duboscqi*, but some comment on this would be helpful in the context of future work. Although similar in outcome the results of their bites may not be identical, so it would be better to work with one species or the other and not to mix and match results when it comes to subsequent infection challenge studies, or to include a full comparison as part of those if resources allow.

 One final comment is that while it is certainly true that the major leishmaniases are known to be sand fly transmitted, there is now evidence that one group of *Leishmania* parasites, those belonging to the subgenus *Mundinia*, may have an alternate vector, biting midges. These insects feed and presumably transmit in a very similar way to phlebotomine sand flies, they are both pool feeders, so whilst some adjustments may be necessary, the approaches being developed here should be largely transferable to this group of parasites.

Is the work clearly and accurately presented and does it cite the current literature?

Yes

If applicable, is the statistical analysis and its interpretation appropriate?

Yes

Are all the source data underlying the results available to ensure full reproducibility?

Yes

Is the study design appropriate and is the work technically sound?

Yes

Are the conclusions drawn adequately supported by the results?

Yes

Are sufficient details of methods and analysis provided to allow replication by others?

Yes

Reviewer Expertise:

Leishmaniasis and sand flies, including life cycle biology of the parasite and transmission by sand fly bite.

I confirm that I have read this submission and believe that I have an appropriate level of expertise to confirm that it is of an acceptable scientific standard.
